# The causality between dietary intake and allergic diseases: A two-sample Mendelian randomization analysis

**DOI:** 10.1097/MD.0000000000047741

**Published:** 2026-03-06

**Authors:** Wenting Liu, Chengxiang Mao, Hanfeng Ji, Fengli Cheng, Tingting Li, Qiqi Li, Changqing Zhao, Yunfang An

**Affiliations:** a Department of Otolaryngology–Head and Neck Surgery, the Second Hospital, Taiyuan, Shanxi, China; b Shanxi Medical University, Taiyuan, Shanxi, China.

**Keywords:** allergic asthma, allergic rhinitis, atopic dermatitis, causality, dietary intake, Mendelian randomization

## Abstract

Allergic diseases are common chronic inflammatory conditions, which can affect multiple organs in severe cases, resulting in complex and varied clinical manifestations. Therefore, their management requires a more comprehensive and long-term strategy. A well-balanced diet is crucial in this context, as it regulates the immune system and improves atopic constitution, making it a key measure in preventing and controlling allergic diseases. Unlike observational studies prone to confounding and reverse causality, Mendelian randomization uses genetic variants as instrumental variables for stronger causal inference. This study employed the two-sample Mendelian randomization to investigate the potential causal relationships between 22 dietary factors and allergic diseases. The primary methods used were the weighted median method, MR-Egger regression, and inverse-variance weighted. To ensure the robustness and accuracy of the results, a series of sensitivity analyses, heterogeneity tests, and pleiotropy assessments were conducted. The study identified 7 dietary factors associated with allergic asthma, atopic dermatitis, and allergic rhinitis. The oily fish (OR: 0.666; 95% CI: 0.468–0.949; *P* = .024), dried fruit (OR: 0.463; 95% CI: 0.307–0.697; *P* = .00023), and cereal intake (OR: 0.595; 95% CI: 0.355–0.998; *P* = .049) was found to have a protective effect against asthma. The fresh fruit (OR: 0.592; 95% CI: 0.384–0.913; *P* = .018), tea (OR: 0.774; 95% CI: 0.603–0.995; *P* = .046), cereal (OR: 0.635; 95% CI: 0.430–0.939; *P* = .023), and processed meat intake (OR: 0.481; 95% CI: 0.294–0.787; *P* = .0036) were protective factors against atopic dermatitis. No significant causal relationships were observed between other dietary factors and these 3 diseases. These findings underscore the critical role of a balanced diet in the prevention and management of allergic diseases and highlight the potential of nutritional interventions in the future control and treatment of these conditions.

## 1. Introduction

Allergic diseases, including allergic asthma (AA), atopic dermatitis (AD), and allergic rhinitis (AR), have seen a sharp increase in prevalence since the late 20th century.^[1,2]^ Surveys indicate that these diseases now affect 40% of the population^[3]^ and have been identified by the World Health Organization (WHO) as one of the 3 major diseases requiring focused research and control in the 21st century, presenting a serious public health challenge.^[4]^ These conditions are primarily driven by an IgE-mediated immune response to environmental allergens, leading to abnormal reactions.^[5]^ In severe cases, they can affect multiple organs, such as the skin (itching, swelling), lungs (coughing, wheezing), and nasal passages (congestion, runny nose, sneezing), resulting in complex clinical symptoms. These conditions not only negatively impact individuals’ income, quality of life, and productivity,^[6]^ but also impose a significant burden on society.^[7]^ Currently, the treatment of allergic diseases largely relies on systemic or topical corticosteroids to alleviate symptoms, but these symptoms often recur, and complications are common.^[8]^ Consequently, there is a growing recognition that prevention is a crucial component of managing allergic diseases, prompting a strategic shift towards focusing on overall dietary patterns.^[9]^ The complex interplay between genetic and environmental factors in allergic diseases results in abnormal immune responses at barrier sites in the body.^[10–12]^ Previous studies have reported that dietary factors are key influencers of immune homeostasis and the development of allergic diseases.^[13]^ Understanding whether dietary modifications benefit patients with allergic diseases is of significant importance to both clinicians and patients.

However, the causal relationship between dietary factors and these 3 allergic diseases remains unclear due to the lack of evidence from randomized controlled trials (RCTs). While RCTs are considered the gold standard for causal inference in epidemiological studies, they are often challenging to conduct because of high costs and ethical constraints.^[14]^ Mendelian randomization (MR), which uses genetic variants as instrumental variables (IVs), offers distinct advantages over other research methods^[15]^ and serves as an effective alternative.^[16]^ In this study, we conducted a series of two-sample Mendelian randomization (TSMR) analyses using summary data from large research institutions to assess whether 22 dietary factors are risk factors for the development of allergic diseases. Clarifying these associations could provide valuable evidence for preventing allergic diseases and developing effective dietary strategies.

## 2. Materials and methods

### 2.1. Research design

**Figure 1.**
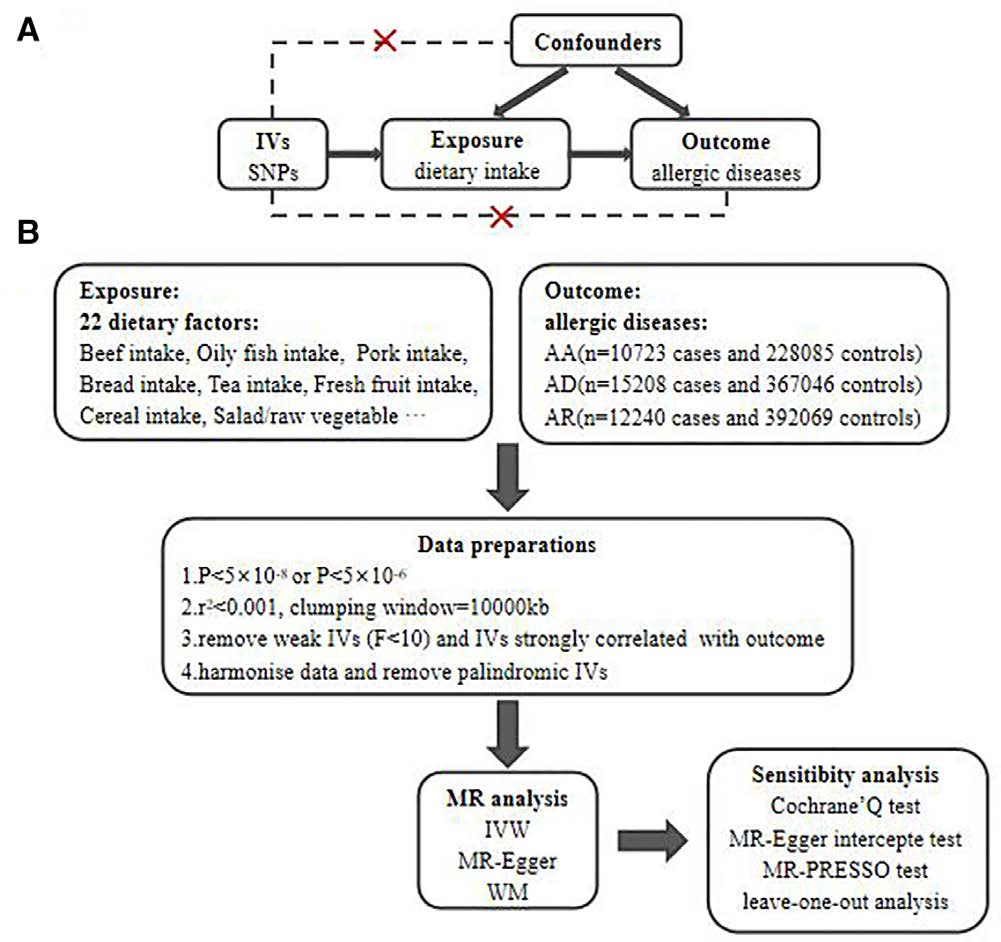
The overview flowchart of hypothesis and schematic design. (A) assumptions of MR. (B) Overview of present MR analyses. AA = allergic asthma, AD = atopic dermatitis, AR = allergic rhinitis, IVs = instrumental variables, IVW = inverse-variance weighted, SNPs = single nucleotide polymorphisms, WM = weighted median.

This study employed a TSMR to investigate the causal relationships between various dietary exposures and allergic diseases (AA, AD, and AR). An overview of the study design is presented in Figure 1. To ensure the validity of the TSMR method, the selected IVs had to meet 3 core assumptions: 1) the genetic variants must be strongly associated with the exposure factors; 2) there is no correlation between genetic variants and confounders; 3) the genetic variants must influence the outcome solely through the exposure factors, with no alternative pathways, thereby avoiding horizontal pleiotropy. Genetic variants that fulfilled these 3 assumptions were incorporated as IVs in the TSMR analysis.^[17]^ This study was exempt from ethics committee approval as the data used were publicly available, anonymized, and de-identified, thus requiring no additional ethical approval.

### 2.2. Data sources and selection of IVs

GWAS data for the 22 dietary exposure factors were sourced from the IEU Open GWAS Project (https://gwas.mrcieu.ac.uk/), with these summary data directly or indirectly extracted from UK Biobank. The dietary data primarily originate from the Benjamin Neale Laboratory (http://www.nealelab.is/uk-biobank/). The dietary factors include meat intake (oily fish intake, non-oily fish intake, bacon intake, processed meat intake, poultry intake, beef intake, pork intake, lamb/mutton intake), fruit intake (fresh fruit intake, dried fruit intake), vegetable intake (salad/raw vegetable intake, cooked vegetable intake), fluid intake (coffee intake, tea intake, alcohol intake frequency, alcoholic drinks per week), staple food intake (bread intake, cereal intake), dairy product intake (cheese intake, yogurt intake), and other dietary habits (whole egg intake, salt added to food) (Table 1). GWAS summary statistics for AA, AD, and AR were obtained from the FinnGen Research (https://r10.finngen.fi/), released in December 2023. The diagnostic criteria for AA were based on ICD-10, with the GWAS statistics encompassing 21,299,655 variant sites from 10,723 cases and 228,085 controls. The diagnostic criteria for AD were based on ICD-8, ICD-9, and ICD-10, with the GWAS statistics including 21,305,750 variant sites from 15,208 cases and 367,046 controls. The diagnostic criteria for AR were based on ICD-9 and ICD-10, with the GWAS statistics covering 21,306,212 variant sites from 12,240 cases and 392,069 controls. For more information on the exposure and outcome datasets (Table S1, Supplemental Digital Content, https://links.lww.com/MD/R468).

**Table 1:** Summary of causality between dietary intake and allergic diseases.

Traits	Dietary intake	SNPs	Method	OR	95% CI	*P*-value
AA	Oily fish intake	57	IVW	0.666	0.468–0.949	.024
			Cochran *Q*			.013
			MR-Egger intercept			.137
			MR-PRESSO			.012
AA	Dried fruit intake	38	IVW	0.463	0.307–0.697	.0002
			Cochran *Q*			.599
			MR-Egger intercept			.036
			MR-PRESSO			.470
AA	Cereal intake	37	IVW	0.595	0.355–0.998	.049
			Cochran *Q*			.001
			MR-Egger intercept			.800
			MR-PRESSO			<.001
AD	Fresh fruit intake	52	IVW	0.592	0.384–0.913	.018
			Cochran *Q*			.149
			MR-Egger intercept			.744
			MR-PRESSO			.142
AD	Tea intake	40	IVW	0.774	0.603–0.995	.046
			Cochran *Q*			.148
			MR-Egger intercept			.905
			MR-PRESSO			.181
AD	Cereal intake	37	IVW	0.635	0.430–0.939	.023
			Cochran *Q*			.018
			MR-Egger intercept			.397
			MR-PRESSO			.048
AD	Processed meat intake	22	IVW	0.481	0.294–0.787	.004
			Cochran *Q*			.029
			MR-Egger intercept			.620
			MR-PRESSO			.027

AA = allergic asthma, AD = atopic dermatitis, CI = confidence interval, IVW = inverse-variance weighted method, OR = odd ratio, SNPs = number of single nucleotide polymorphism.

To ensure robustness and an adequate number of IVs, we applied stringent thresholds of *P* < 5 × 10^−8^ or *P* < 5 × 10^−6^. Additionally, we set the linkage disequilibrium correlation coefficient to *r*^2^ < 0.001, with clumping window >10,000 kb, to filter single nucleotide polymorphisms (SNPs) closely associated with the exposures. During the harmonization of exposure and outcome statistics, we excluded palindromic and incompatible SNPs, as well as exposure-related SNPs that could not be matched in the GWAS outcome data. To avoid horizontal pleiotropy, we used PhenoScanner V2 to exclude IVs associated with risk factors for allergic diseases.^[18]^ Furthermore, we calculated the *F*-statistic to ensure a strong association between the IVs and exposures, with an *F*-statistic >10 generally considered indicative of sufficient strength.^[19]^

### 2.3. Data analysis

We employed the inverse-variance weighted (IVW) method as the primary approach for causal inference in TSMR analysis.^[20,21]^ To provide broader confidence intervals and enhance the robustness of our findings,^[22]^ we also applied supplementary methods, including weighted median method (WM) and MR-Egger regression.^[23]^ The weighted median method accommodates up to 50% invalid IVs, while the MR-Egger method permits all IVs to be invalid, increasing the reliability of the results when all 3 models yield consistent outcomes. Heterogeneity within the IVW model was evaluated using Cochran *Q* test (*P* < .05 indicates heterogeneity). Nonetheless, the presence of heterogeneity does not necessarily invalidate the IVW model. We employed MR-Egger regression and the MR-PRESSO test to detect and exclude outliers in order to address possible horizontal pleiotropy.^[24,25]^ After removing outliers, we repeated the MR analysis. Additionally, we performed a leave-one-out sensitivity analysis to evaluate the influence of each SNP on the stability of the results. All analyses were conducted using the TwoSampleMR package in R software (version 4.2.2).^[26]^

## 3. Results

**Figure 2.**
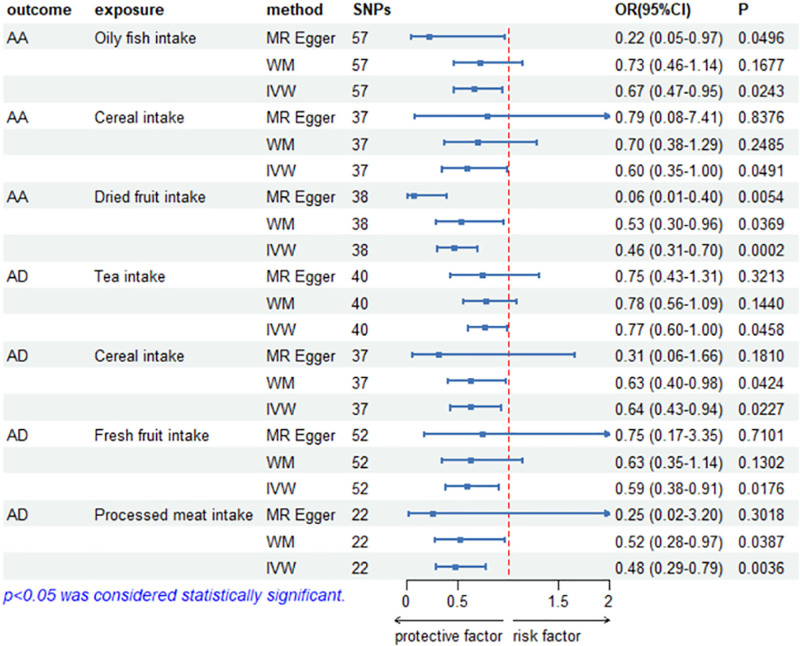
Forest plot of the causality between cross-validated dietary intake with the risks of allergic diseases. AA = allergic asthma, AD = atopic dermatitis, IVW = inverse-variance weighted, SNPs = single nucleotide polymorphisms, WM = weighted median.

Based on the criteria for selecting IVs, the number of SNPs used in this study ranged from 3 to 98, with 0 to 4 outliers identified across different exposures. All SNPs had *F*-statistics >10, indicating the absence of weak IVs. We utilized 3 methods to analyze the causal relationships between 22 dietary exposure factors and the 3 allergic diseases (Tables S2–S4, Supplemental Digital Content, https://links.lww.com/MD/R468). Potential causal relationships were identified for 4 dietary factors with AA, 6 dietary factors with AD, and 1 dietary factor with AR, using one or more methods. After conducting sensitivity analyses and comprehensive validation, we focused primarily on 3 dietary factors associated with AA and 4 dietary factors associated with AD (Table 1, Fig. 2), with the results demonstrating robustness and reliability.

### 3.1. Allergic asthma

TSMR identified causal relationships between 4 dietary factors and AA. Further analysis confirmed consistent results for 3 of these factors. Specifically, the intake of oily fish (outlier-corrected OR: 0.666; 95% CI: 0.468–0.949; *P* = .024), cereal (outlier-corrected OR: 0.595; 95% CI: 0.355–0.998; *P* = .049), and dried fruit (outlier-corrected OR: 0.463; 95% CI: 0.307–0.697; *P* = .00023) were all found to have protective effects against AA, while poultry intake (OR: 5.492; 95% CI: 1.071–28.155; *P* = .041) was associated with an increased risk of AA.

The MR-Egger method was used to further validate the intake of oily fish (OR: 0.220; 95% CI: 0.051–0.965; *P* = .04961) and dried fruit (OR: 0.065; 95% CI: 0.011–0.397; *P* = .005). The weighted median model was applied to validate poultry intake (OR: 6.772; 95% CI: 1.252–36.634; *P* = .026) and dried fruit intake (OR: 0.534; 95% CI: 0.296–0.962; *P* = .0369). The MR-Egger method produced results for poultry intake (OR: 4.01E−09) that were in the opposite direction to those obtained with the IVW method, and the analysis, after expanding the *P*-value threshold to include more SNPs, yielded consistent results (OR: 0.663) with no significant correlation (IVW *P* = .820). Although heterogeneity was detected in most exposures (Cochran *Q* test *P* < .05), the random effects model was used by default to estimate MR effect sizes. The MR-Egger intercept results indicated no directional pleiotropy for exposures other than dried fruit intake. The leave-one-out analysis confirmed the robustness of the causal relationships for the positive results. However, it is noteworthy that the *P*-values for dried fruit intake were <.05 across all 3 methods, and the MR-PRESSO global test showed *P* = .47, suggesting that the influence of this factor cannot be disregarded.

### 3.2. Atopic dermatitis

The IVW method identified causal relationships between 6 dietary factors and AD. Further analysis confirmed consistent results for 4 of these factors. Specifically, tea intake (OR: 0.774; 95% CI: 0.603–0.995; *P* = .046), cereal intake (outlier-corrected OR: 0.635; 95% CI: 0.430–0.939; *P* = .023), fresh fruit intake (OR: 0.592; 95% CI: 0.384–0.913; *P* = .018), and processed meat intake (outlier-corrected OR: 0.481; 95% CI: 0.294–0.787; *P* = .0036) all demonstrated protective effects against AD. In contrast, salad/raw vegetable intake (outlier-corrected OR: 1.960; 95% CI: 1.005–3.827; *P* = .048) and cooked vegetable intake (outlier-corrected OR: 1.956; 95% CI: 1.043–3.669; *P* = .037) was associated with a higher risk of AD.

We reached the same conclusions using the weighted median model, which showed that processed meat intake (OR: 0.521; 95% CI: 0.280–0.967; *P* = .039) and cereal intake (OR: 0.630; 95% CI: 0.403–0.984; *P* = .042) had protective effects. In contrast, the results for cooked vegetable intake in the MR-Egger method were in the opposite direction (OR: 0.029) compared to the IVW method. After expanding the *P*-value threshold to include more SNPs, no significant correlation was found (IVW *P* = .357). For salad/raw vegetable intake, the confidence interval in the MR-Egger analysis was excessively wide (95% CI: 0.071–44.898), and no significant correlation was observed after expanding the range (*P* = .507). Heterogeneity was detected in the analyses of processed meat and cereal intake (Cochran *Q* test *P* < .05), but the MR-Egger intercept did not reveal directional pleiotropy. Furthermore, the leave-one-out analysis confirmed the robustness of the causal relationships for the positive results.

### 3.3. Allergic rhinitis

The IVW method identified a causal relationship between dietary factors and AR, specifically with cooked vegetable intake (OR: 2.307; 95% CI: 1.008–5.279; *P* = .048). However, in the MR-Egger analysis, the confidence interval for cooked vegetable intake was excessively wide (95% CI: 0.0002–31,343.212), and after expanding the range, no significant correlation was observed (*P* = .352).

## 4. Discussion

To date, numerous studies have investigated the relationship between dietary factors and allergic diseases.^[27,28]^ However, research exploring the causal links between diet and allergic diseases from a genetic perspective remains scarce. This study employed a TSMR design, utilizing GWAS data to address this question. Our findings on the impact of oily fish intake on allergic diseases align with previous studies. Papamichael et al^[29]^ conducted a 6-month randomized controlled trial comparing the effects of supplementing 2 meals per week with 150 g of cooked oily fish versus a regular diet on pulmonary function and bronchial inflammation in children with mild asthma (ages 5–12). The results suggested that oily fish, as part of a Mediterranean diet, could be a potential strategy for reducing airway inflammation in children with asthma. Similarly, Mickleborough et al^[30]^ found through a randomized, double-blind, crossover study that fish oil supplements could be a potential non-pharmacological intervention for exercise-induced bronchoconstriction in athletes. Our findings suggest that only the oily fish intake serves as a protective factor against AA, with no direct link found between non-oily fish intake and the illness. The increased concentration of omega-3 polyunsaturated fatty acids (PUFAs) in oily fish, specifically eicosapentaenoic acid (EPA) and docosahexaenoic acid (DHA), may be responsible for this protective impact.^[31]^ PUFAs in oily fish have been associated with a reduced risk of allergic diseases,^[32,33]^ as further corroborated by a randomized controlled trial conducted by Nagakura et al^[34]^ Our study provides genetic evidence supporting a potential protective role of oily fish against AA, which may inform future research directions for prevention strategies.

It is well established that airways are susceptible to oxidative damage, with oxidants capable of inducing the release of pro-inflammatory mediators, including cytokines and chemokines, thereby triggering asthma symptoms.^[35]^ Research has shown that fresh fruits, rich in antioxidants, can help prevent allergic diseases.^[36]^ The primary mechanism of antioxidants in anti-allergic therapy is their ability to block inducible nitric oxide synthase.^[37]^ Previous studies have found a negative correlation between fruit intake and the occurrence of asthma symptoms.^[38,39]^ A prospective study in the UK reported that a sustained high intake of fruits could reduce the risk of asthma and AD in children.^[40]^ Dried fruits, which contain various bioactive compounds such as micronutrients, fiber, and antioxidants – particularly phenolic compounds – offer concentrated polyphenol content through the drying process. This concentration endows dried fruits with significant antioxidant capacity, which can promote blood circulation, enhance gut health, reduce oxidative stress, and lower the risk of disease.^[41]^ A MR study also indicated that dried fruit intake is a protective factor against asthma.^[42]^

Studies have shown that a Mediterranean diet, which emphasizes cereals, fruits, and vegetables, has a protective effect against allergic diseases. Similarly, vegetarian diets have demonstrated potential benefits in reducing allergic symptoms.^[28]^ Meyer et al^[43]^ identified a dietary pattern associated with lower levels of inflammatory markers through Reduced Rank Regression analysis, highlighting cereals as part of a healthy diet linked to lower inflammatory markers and a reduced risk of coronary heart disease. Our study found that cereal intake is a protective factor against asthma and AD; however, no causal relationship was observed between cereal intake and AR. This discrepancy may require further observational studies and more sophisticated MR research to explore in greater depth.

Tea has been found to possess anti-allergic properties, with polyphenols likely acting as “mast cell membrane stabilizers.”^[44]^ Inhibiting relevant transcription factors, lowering IgE and histamine levels, decreasing FcεRI expression, and altering the balance of Th1/Th2/Th17/Treg cells are the main ways that tea has anti-allergic effects.^[45]^ A randomized controlled trial by Abe et al^[46]^ demonstrated that fig leaf tea could serve as a natural and effective treatment option for AD, offering a safe alternative to current therapies. Our findings lend further genetic support to the potential role of tea components in modulating allergic pathways.

Previous studies have yielded mixed conclusions regarding the allergenicity of different types of processed meats. The meat industry commonly uses various food additives in the production of these products, some of which can cause severe allergic reactions.^[47]^ Li et al^[48]^ conducted a study involving 15,062 participants and found that those who consumed processed meats 1 to 3 times per month and 1 to 3 times per week had a 29% and 44% increased risk of AD, respectively, compared to those who rarely ate processed meats. In contrast, Xie et al^[49]^ discovered that using lactic acid bacteria as a fermenting agent – due to its amine oxidase activity and ability to inhibit the growth of Enterobacteriaceae – is beneficial for controlling biogenic amines in fermented sausages. Some meats fermented with Lactic Acid Bacterium have been shown to alleviate AR. This microorganism could regulate immunity through the microbiota–gut–brain axis signaling, suppressing the activity of immune cells (e.g. helper T cells) and increasing the population of Tregs, thereby significantly improving inflammatory responses.^[50]^ These studies suggest that the relationship between processed meats and allergic diseases remains contentious. Our study identified processed meat intake as a protective factor against AD. In summary, the impact of processed meats on allergic diseases is complex, and further analysis is needed to fully understand these mechanisms.

Interestingly, this study did not find potential causal relationships between vegetables, dairy products, and other dietary factors and allergic diseases, which contrasts with previous research findings.^[51,52]^ Several reasons may explain this discrepancy. First, some studies might have been influenced by potential confounding factors. Second, while a causal relationship might exist between these factors and allergic diseases, MR studies, as an emerging research method, are limited by issues such as population stratification and developmental compensation, which could prevent the detection of these relationships. Therefore, future research should focus on conducting more studies, designing more sophisticated MR analyses, such as nonlinear or multivariable MR analyses, and improving correction and estimation methods to uncover deeper connections.

It is noteworthy that growing attention is being paid to the role of daily diet in allergic diseases. The primary aim of this study was to highlight specific dietary factors as potential candidates for further investigation in the context of allergic disease prevention, and provide evidence-based dietary recommendations. Diet plays a critical role in the onset and severity of allergic diseases by regulating tissue and immune homeostasis. Research has shown that various dietary components, including vitamins A, D, and E, minerals such as zinc and iron, dietary fiber, fatty acids, and phytochemicals, contribute to the prevention or treatment of allergic diseases by inhibiting type 2 inflammation. Additionally, dietary factors may exert their effects through the gut microbiota, as the intake of different foods can influence the composition of gut bacteria, thereby affecting nutrient metabolism.^[28]^ An increasing number of dietary patterns have also been found to be associated with allergic diseases. Studies have demonstrated that plant-based diets, particularly vegan diets, are beneficial in treating inflammatory skin diseases, including AD.^[53,54]^ The Mediterranean diet, which emphasizes vegetables, fruits, fish, whole grains, legumes, and olive oil, also positively impacts allergic diseases.^[55]^ Therefore, exploring the anti-allergic potential of dietary phytochemicals and their metabolites is a promising direction for basic research, and scientifically investigating the integration of these dietary components into broader nutritional frameworks represents a key area for future research in the management of allergic diseases.

Additionally, this study has certain strengths and limitations. One of its strengths is that it is based on large-sample, publicly available GWAS data, which allows for the exploration of causal relationships between diet and allergic diseases at the genetic level. This approach offers advantages such as a large-sample size and the avoidance of ethical constraints. By using genetic variants as IVs, MR helps mitigate biases arising from confounding, measurement error, and reverse causation, thereby enhancing the validity and accuracy of the study’s findings. However, this study also has some limitations. First, the GWAS data used were derived exclusively from European populations, so future research should include different dietary patterns and global populations. Second, this study considered the exposure factors as a whole and did not conduct a classified analysis of specific eating habits and frequencies, nor could it distinguish the effects of different dietary combinations. More sophisticated MR studies are needed in the future to conduct in-depth analyses. Third, although we did not identify potential causal effects of vegetables, dairy products, and other dietary factors on allergic diseases, measurement and definition issues cannot be ruled out, necessitating further analysis to confirm these findings.

The results obtained from this study provide genetic insights into the associations between specific dietary factors and allergic diseases. They serve as an important reference for generating hypotheses for future mechanistic studies, public health research, and the eventual development of evidence-based nutritional strategies, which may contribute to reducing the burden of allergic diseases.

## 5. Conclusion

**Figure 3.**
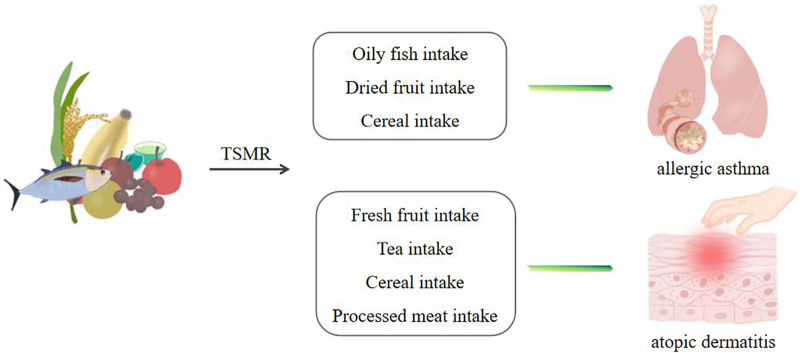
Dietary intake associated with allergic diseases identified by the current MR analysis. The green arrow indicates that the dietary intake is the protective. MR = Mendelian randomization, TSMR = two-sample Mendelian randomization.

In summary, this study explored the potential causal relationships between genetically predicted intake of 22 common dietary factors and allergic diseases. The findings suggest that increased intake of oily fish, dried fruit, and cereal may reduce the risk of AA, while higher intake of fresh fruit, tea, cereal, and processed meat is associated with a lower risk of AD (Fig. 3). These results provide valuable insights for the prevention and treatment of allergic diseases and serve as an important reference for future studies, public health policy development, and clinical prevention strategies. Future research will investigate a broader range of dietary options and their underlying mechanisms to support evidence-based strategies that may help reduce the progression of allergic diseases.

## Author contributions

**Conceptualization:** Changqing Zhao, Yunfang An.

**Data curation:** Wenting Liu.

**Formal analysis:** Fengli Cheng, Tingting Li.

**Funding acquisition:** Fengli Cheng, Changqing Zhao, Yunfang An.

**Methodology:** Wenting Liu.

**Supervision:** Hanfeng Ji, Fengli Cheng, Tingting Li, Qiqi Li, Changqing Zhao, Yunfang An.

**Validation:** Hanfeng Ji, Fengli Cheng, Tingting Li, Qiqi Li, Changqing Zhao, Yunfang An.

**Writing – original draft:** Wenting Liu, Hanfeng Ji.

**Writing – review & editing:** Wenting Liu, Chengxiang Mao, Hanfeng Ji, Fengli Cheng, Tingting Li, Qiqi Li, Changqing Zhao, Yunfang An.

## Supplementary Material


